# Diagnostic and Prognostic Accuracy of Aortic Valve Calcium Scoring in Patients With Moderate-to-Severe Aortic Stenosis

**DOI:** 10.3389/fcvm.2021.673519

**Published:** 2021-05-17

**Authors:** Jamila Boulif, Alisson Slimani, Siham Lazam, Christophe de Meester, Sophie Piérard, Agnès Pasquet, Anne-Catherine Pouleur, David Vancraeynest, Gébrine El Khoury, Laurent de Kerchove, Bernhard L. Gerber, Jean-Louis Vanoverschelde

**Affiliations:** ^1^Pôle de Recherche Cardiovasculaire, Institut de Recherche Expérimentale et Clinique, Université Catholique de Louvain, Brussels, Belgium; ^2^Divisions of Cardiology and Cardiothoracic Surgery, Cliniques Universitaires Saint-Luc, Brussels, Belgium

**Keywords:** aortic stenosis, low gradient, aortic valve calcium, computed tomgraphy, outcome

## Abstract

**Background:** Assessing the true severity of aortic stenosis (AS) remains a challenge, particularly when echocardiography yields discordant results. Recent European and American guidelines recommend measuring aortic valve calcium (AVC) by multidetector row computed tomography (MDCT) to improve this assessment.

**Aim:** To define, using a standardized MDCT scanning protocol, the optimal AVC load criteria for truly severe AS in patients with concordant echocardiographic findings, to establish the ability of these criteria to predict clinical outcomes, and to investigate their ability to delineate truly severe AS in patients with discordant echocardiographic AS grading.

**Methods and Results:** Two hundred and sixty-six patients with moderate-to-severe AS and normal LVEF prospectively underwent MDCT and Doppler-echocardiography to assess AS severity. In patients with concordant AS grading, ROC analysis identified optimal cut-off values for diagnosing severe AS using different AVC load criteria. In these patients, 4-year event-free survival was better with low AVC load (60–63%) by these criteria than with high AVC load (23–26%, log rank *p* < 0.001). Patients with discordant AS grading had higher AVC load than those with moderate AS but lower AVC load than those with severe high-gradient AS. Between 36 and 55% of patients with severe LG-AS met AVC load criteria for severe AS. Although AVC load predicted outcome in these patients as well, its prognostic impact was less than in patients with concordant AS grading.

**Conclusions:** Assessment of AVC load accurately identifies truly severe AS and provides powerful prognostic information. Our data further indicate that patients with discordant AS grading consist in a heterogenous group, as evidenced by their large range of AVC load. MDCT allows to differentiate between truly severe and pseudo-severe AS in this population as well, although the prognostic implications thereof are less pronounced than in patients with concordant AS grading.

## Introduction

Several recent retrospective studies have indicated that, in elderly patients and particularly in elderly women with severe aortic stenosis (AS), physicians are frequently confronted with lower than expected mean transvalvular gradients, even in the presence of a preserved left ventricular ejection fraction (LVEF) (1–5). To differentiate this new form of severe AS from the classical “low flow (LF)—low gradient” (LG) form seen in patients with LV dysfunction (1), the term “paradoxical LG-AS” was recently proposed (2, 3).

There is considerable debate as to the clinical significance of severe paradoxical LG-AS. Because it is frequently associated with concentric LV remodeling (5), low transvalvular flow rates (2), increased interstitial fibrosis (6), reduced LV long-axis function (5, 7), and guarded prognosis (2, 4, 8–10), several authors have hypothesized it could represent a more advanced form of severe AS. On the other hand, the results of recent natural history studies have indicated that severe paradoxical LG-AS usually evolves into severe high gradient (HG)-AS overtime (11–13) and that its clinical outcome resembles that of moderate AS (12, 14), thus challenging the former hypothesis. It was further suggested that severe paradoxical LG-AS could be an intermediary stage between moderate AS and severe HG-AS (11).

To get further insight into the pathophysiology of this challenging condition, an alternate method for assessing AS severity is highly desirable. We and others have previously shown that aortic valve calcification (AVC) load is a fundamental marker of the severity of the aortic valve (AV) lesions seen in “degenerative” AS and that it can be accurately quantified by use of X-Ray computed tomography modalities, such as Electron Beam Computed Tomography (EBCT) and Multidetector Computed Tomography (MDCT) (15, 16). Based on these observations, the most recent European Society of Cardiology (ESC) guidelines have recommended to use this approach to delineate the severity of AS in patients with discordant grading by echocardiography (17), and proposed specific AVC load thresholds to be used for diagnosing truly severe AS in this setting. Unfortunately, the proposed thresholds were derived from a single multicenter study, which used a wide variety of scanning protocols (18–20), some of which have been shown to significantly affect the resulting AVC load values. Accordingly, the aims of the present study were to define, using a standardized MDCT scanning protocol, the optimal AVC load criteria for truly severe AS in patients with concordant echocardiographic findings, to establish the potential of these criteria to predict clinical outcomes and to investigate their ability to delineate truly severe AS in patients with discordant echocardiographic AS grading.

## Methods

### Patients' Population

**Figure 1:**
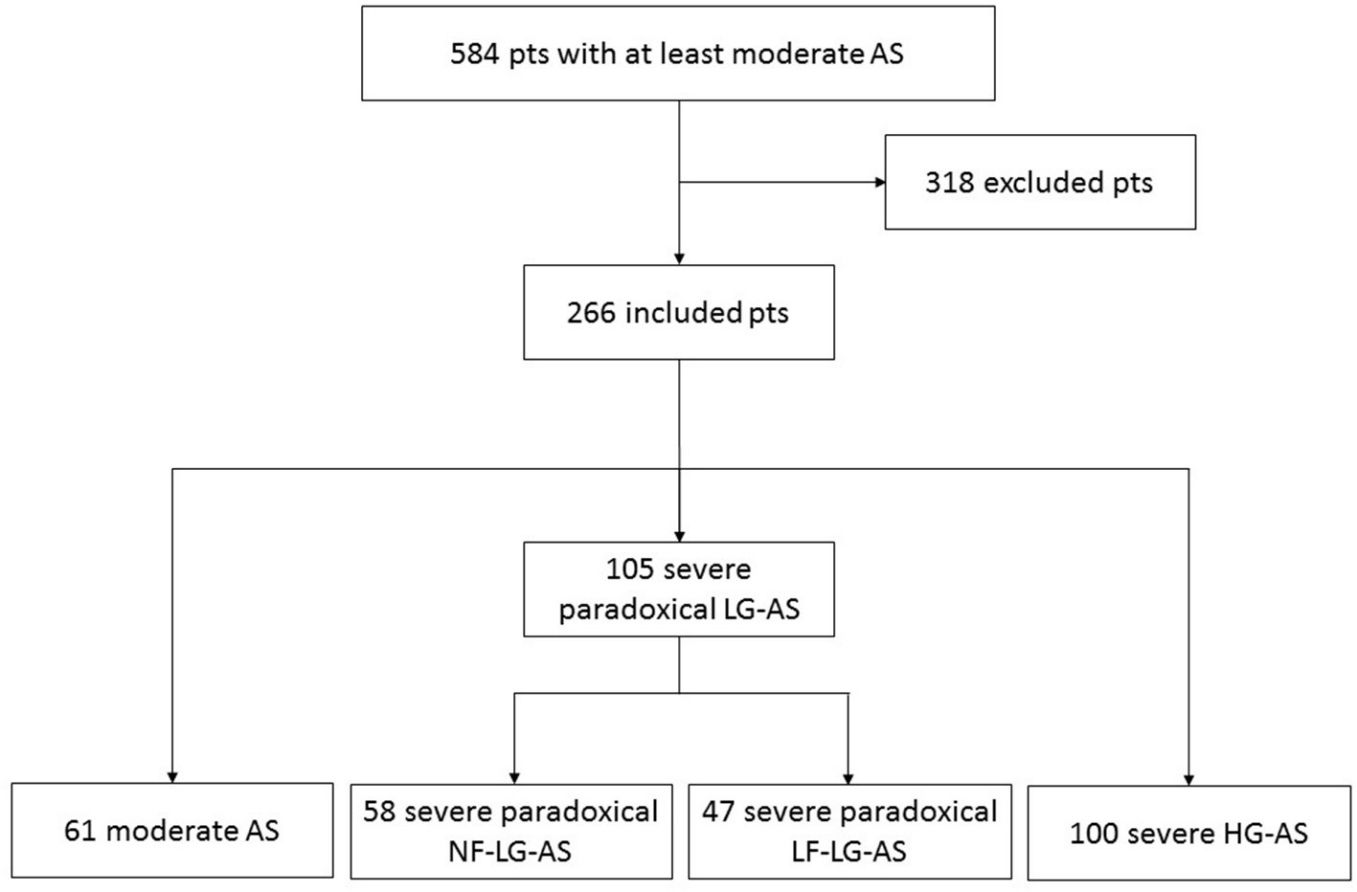
Flow chart of the study population. Pts, patients; AS, aortic stenosis; NF, normal flow; LF, low flow; LG, low gradient; HG, high gradient.

Between February 1st, 2013 and August 31th, 2015, 584 consecutive patients with LVEF > 50% and at least moderate native AS, defined as an effective orifice area (EOA) < 1.5 cm^2^ and an indexed EOA (EOAi) < 0.9 cm^2^/m^2^ by transthoracic echocardiography were prospectively identified in the valvular Clinic of the Cliniques Universitaires St-Luc and approached for inclusion in the IRB approved study (2014/29Nov/560). Patients were included into the study after giving written informed consent. Patients with rheumatic AS, LV dysfunction, more than mild aortic regurgitation or mitral valve disease, poor quality of echocardiographic images or a life expectancy < 1 year in the absence of severe AS were not considered for inclusion. The final study population consisted of 266 patients (Figure 1). After consenting to participate into the study, all patients underwent a comprehensive echocardiographic assessment of their AS and an MDCT examination within 10 ± 19 days of their echocardiographic examination.

### Doppler Echocardiography Measurements

Echocardiographic data were obtained with commercially available ultrasound systems (IE33 or EPIC, Philps Medical System Andover, MA) conducted by experienced sonographers. All patients underwent a comprehensive examination, including M-mode, 2-dimensional and Doppler examinations according to ASE/EACVI recommendations.

For assessment of AS, multiple transducer positions were systematically used to record peak aortic jet velocities. The left ventricular outflow tract (LVOT) diameter was obtained from the parasternal long-axis view in mid-systole, parallel to the valve plane and immediately adjacent to the aortic leaflet insertion into the annulus. The LVOT velocity was recorded from the apical window by placing the pulsed-wave-Doppler sample volume in the LVOT, proximal to the aortic valve. Proper positioning of the sample volume was ensured by verifying the presence of smooth spectral velocity curves associated with an aortic valve closing click. Care was taken to optimize the ultrasound beam—blood flow alignment and to avoid sampling in the transvalvular jet or the proximal flow convergence region by excluding velocity curves with spectral broadening at peak ejection. The maximal velocity across the aortic valve was measured with continuous-wave Doppler from multiple positions (apical, right parasternal, suprasternal, and subxyphoidal). The highest velocity signal was used to calculate peak and mean gradients. The EOA was calculated by use of the continuity equation, assuming that the LVOT area had a circular shape. LV volumes and LVEF were calculated by use of the biplane Simpson method and left atrial volume using biplane area-length method. In case of atrial fibrillation, 5–10 consecutive beats were systematically averaged.

Severe AS was defined as an indexed EOAi < 0.6 cm^2^/m^2^ and was further stratified into subgroups with high and paradoxically low transvalvular gradients, respectively, in the presence of a mean transvalvular gradient (MPG) ≥ and < 40 mmHg. On the basis of EOAi and MPG, patients were categorized in 3 groups: 2 groups with concordant AS grading (moderate AS with an EOAi > 0.6 cm^2^/m^2^ and a MPG < 40 mm Hg and severe high gradient AS with EOAi ≤ 0.6 cm^2^/m^2^ and a MPG ≥ 40 mm Hg) and 1 group with discordant AS grading (severe paradoxical LG-AS with an EOAi < 0.6 cm^2^/m^2^ and MPG < 40 mmHg). Patients with severe paradoxical LG-AS were further stratified into subgroups with low flow (LF) and normal flow (NF), respectively, in the presence of an indexed stroke volume < 35 or ≥ 35 mL/m^2^.

### Multidetector Computed Tomography Measurements

All MDCT examinations were performed by use of a helical 256-slice CT scanner (Brillance ICT, Philips Healthcare, Cleveland, Ohio, USA). Acquisition parameters were set as follows: tube potential of 120 kV, tube current of 250 mA, gantry rotation time of 330 ms, detector configuration of 32 × 0.625 mm, and pitch of 0.14–0.18. Contiguous non-overlapping slices of 2.5 mm were acquired in a craniocaudal direction during inspiratory breathhold and using prospective ECG-triggering at 75% of R-R interval and a CB filter. No contrast enhancement was needed and no beta-blocker was administered for the purpose of the examination. The average of the total estimated effective radiation dose per CT scan was 0.89 ± 0.08 mGy and the average dose-length product was 64 ± 6 mGy.cm.

All measurements of AVC were performed on dedicated workstations using a validated commercially available software (heartbeat calcium scoring; Philips Medical Systems). Calcifications were identified by using a threshold of CT attenuation of 130 Hounsfield Units (HU), based on Agatston scoring method (21). Measurements were made in the axial view by a single investigator who identified the calcifications corresponding to the aortic valve leaflets. For this purpose, the aortic valve was visualized in multiple planes, including cross-sectional valve plane, to accurately exclude contiguous calcium in the mitral valve annulus, the aortic wall and the coronary arteries. The Agatston score was reported as Agatston units (AU). AVC index was computed as the Agatston score divided by BSA and AVC density as Agatston score indexed to the LVOT cross sectional area (measured from echocardiographic data). The accuracy of our measurements was demonstrated in a previous study by anatomical validation, using *in vivo* (*r* = 0.86, *p* < 0.001) and *ex vivo* (*r* = 0.93, *p* < 0.001) AVC measurements (16).

### Outcome

Follow-up events were obtained for all patients between September and December 2018 by recalling physicians, cardiologists or patients themselves. Causes of death were established by autopsy records if the patient died in hospital, and otherwise by the referring physician. The primary outcome was the time to first event of death or aortic valve replacement, including both open surgical and transcatheter procedures. Decisions to proceed to aortic valve replacement were made according to international clinical guidelines and independently of MDCT calcium scoring, the results of which were not made available to the multidisciplinary discussion team. Patients in whom a decision to refer to aortic valve replacement had been made prior to the CT calcium scoring were excluded from the outcome analyses (*n* = 78).

### Statistical Methods

All analyses were performed using the SPSS v19.0 (SPSS Inc., IBM, Chicago, IL) software. Normality was assessed by use of the Kolmogorov Smirnov-test. Continuous variables were expressed as mean ± 1 SD and were compared among groups using ANOVA when normally distributed or else using the Kruskall-Wallis-test. Individual differences among groups were compared *post-hoc* using Tukey-Kramers-test for normally distributed data with equal variances, the Games–Howell-test for normally distributed data with unequal variances and the Mann-Whitney *U*-tests (with Bonferroni correction for multiple comparisons) for non-normally distributed data. Categorical variables were expressed as counts and percentages and were compared among groups using χ^2^ or the Fisher exact-test. In patients with concordant echocardiographic data, receiver operator curves were used to assess AVC load thresholds and to identify the optimum thresholds for severe AS. Their predictive value was evaluated by computing the area under the ROC curves. Kaplan–Meier curves and Cox proportional hazards regression analyses were used to determine the ability of these AVC load thresholds to predict adverse clinical events. Where appropriate, collinearity of variables was assessed before inclusion in the multivariable model. All tests were two-sided and a *p*-value of < 0.05 was considered indicative of a statistically significant difference.

## Results

### Baseline Clinical, Hemodynamic, and Echocardiographic Characteristics

The final study population consisted of 266 patients [151 men (57%), mean age: 77 ± 10 years] of which, 61 (22.9%) presented with moderate AS, 58 (22%) with severe paradoxical NF-LG-AS, 47 (18%) with severe paradoxical LF-LG-AS and 100 (38%) with severe HG-AS (Figure 1). The clinical and demographic characteristics of these 4 groups are shown in Table 1 and their echocardiographic parameters in Table 2. Overall, the clinical, demographic and echocardiographic characteristics were similar between groups, except for the glomerular filtration rate, which was significantly higher in patients with moderate AS than in those with severe paradoxical NF-LG-AS and atrial fibrillation, which was more prevalent in patients with severe paradoxical LF-LG-AS than in those with severe HG-AS. In addition, patients with moderate AS had higher prevalence of hypertension. Patients with severe paradoxical NF-LG-AS also exhibited larger EOAis than the other severe AS groups whereas severe paradoxical LF-LG-AS displayed lower LV volume indexes than the other groups.

**Table 1:** Baseline demographic and clinical characteristics.

	**MAS**	**Severe paradoxical**	**Severe paradoxical**	**Severe HG-AS**	***P-*value**
	**(*n* = 61)**	**NF-LG-AS (*n* = 58)**	**LF-LG-AS (*n* = 47)**	**(*n* = 100)**	
Age, y	79 ± 10	76 ± 10	77 ± 10	76 ± 10	0.52
Male sex, *n* (%)	31 (51)	35 (60)	30 (64)	55 (55)	0.519
Body surface area, kg/m^2^	1.77 ± 0.21	1.84 ± 0.20	1.83 ± 0.17	1.84 ± 0.23	0.191
Heart rate, beat/min	69 ± 12	68 ± 13	73 ± 18	67 ± 12	0.173
Systemic hypertension, *n* (%)	56 (92)^†^^§^	40 (69)^*^	38 (81)	72 (72)^*^	0.009
Dyslipidemia, *n* (%)	37 (61)	42 (72)	35 (74)	67 (67)	0.394
Diabetes, *n* (%)	11 (18)	12 (21)	13 (28)	18 (18)	0.552
Smoking, *n* (%)	24 (39)	24 (41)	22 (47)	39 (39)	0.827
Family history, *n* (%)	9 (15)	11 (19)	10 (22)	15 (15)	0.736
Coronary artery disease, *n* (%)	25 (41)	25 (43)	28 (60)	41 (41)	0.169
Atrial fibrillation, %	13 (21)	13 (22)	18 (38)^§^	17 (17)^‡^	0.039
GFR, mL/min	75 ± 32^†^	61 ± 27^*^	64 ± 20	63 ± 28	0.034
NYHA class III to IV, *n* (%)	15 (25)	14 (24)	14 (30)	28 (28)	0.646
Angina, *n* (%)	17 (28)	18 (31)	15 (32)	28 (28)	0.944
Syncope, *n* (%)	3 (5)	3 (5)	7 (15)	9 (9)	0.219

*AS, aortic stenosis; GFR, glomerular filtration rate; MAS, moderate AS; HG, high gradient; LF, low flow; LG, low gradient; NF, normal flow; NYHA, New York Heart Association*.

* *p < 0.05 vs. MAS;*

† *p < 0.05 vs. severe paradoxical NF-LG-AS;*

‡ *p < 0.05 vs. severe paradoxical LF-LG-AS;*

§ *p < 0.05 vs. severe HG-AS*.

**Table 2:** Baseline echocardiographic characteristics.

	**MAS**	**Severe paradoxical**	**Severe paradoxical**	**Severe HG-AS**	***P-*value**
	**(*n* = 61)**	**NF-LG-AS (*n* = 58)**	**LF-LG-AS (*n* = 47)**	**(*n* = 100)**	
Mean transvalvular flow rate, mL/s	244 ± 44^‡^	240 ± 41^‡^	167 ± 36^*^^†^^§^	233 ± 57^‡^	<0.001
Peak velocity, cm/s	270 ± 45^†^^‡^^§^	360 ± 29^*^^‡^^§^	325 ± 51^*^^†^^§^	460 ± 52^*^^†^^‡^	<0.001
Mean gradient, mmHg	18 ± 6^†^^‡^^§^	31 ± 4^*^^‡^^§^	25 ± 8^*^^†^^§^	53 ± 13^*^^†^^‡^	<0.001
EOA, cm^2^	1.28 ± 0.16^†^^‡^^§^	0.91 ± 0.15^*^^‡^^§^	0.74 ± 0.19^*^^†^	0.68 ± 0.16^*^^†^	<0.001
Indexed EOA, cm^2^/m^2^	0.73 ± 0.10^†^^‡^^§^	0.50 ± 0.02^*^^‡^^§^	0.40 ± 0.09^*^^†^	0.37 ± 0.08^*^^†^	<0.001
Indexed LVEDV, mL/m^2^	60 ± 15^‡^	56 ± 15	50 ± 14^*^^§^	61 ± 15^‡^	0.974
LV ejection fraction, %	61 ± 7	60 ± 7	58 ± 6	59 ± 6	0.152
Indexed LV stroke volume, mL/m^2^	45 ± 10^‡^	43 ± 6^‡^	28 ± 5^*^^†^^§^	42 ± 9^‡^	<0.001
Indexed LA volume, mL/m^2^	72 ± 34	61 ± 28^§^	72 ± 40	76 ± 31^†^	0.064
LVOT diameter, cm	2.1 ± 0.2^§^	2.1 ± 0.2^§^	2.0 ± 0.2^*^^†^	2.0 ± 0.2	<0.001
A. Fib., *n* (%)	7 (11)^‡^	3 (5)^‡^	13 (28)^*^^†^^§^	6 (6)^‡^	<0.001

*AS, aortic stenosis; EOA, effective orifice area; LA, left atrium; LV, left ventricle; LVEDV, left ventricular end-diastolic volume; LVOT, left ventricle outflow tract; MAS, moderate AS; HG, high gradient; LF, low flow, LG, low gradient; NF, normal flow, TR, tricuspid regurgitation*.

* *p < 0.05 vs. MAS;*

† *p < 0.05 vs. severe paradoxical NF-LG-AS;*

‡ *p < 0.05 vs. severe paradoxical LF-LG-AS;*

§ *p < 0.05 vs. severe HG-AS*.

### AVC Load in the Different AS Groups

**Figure 2:**
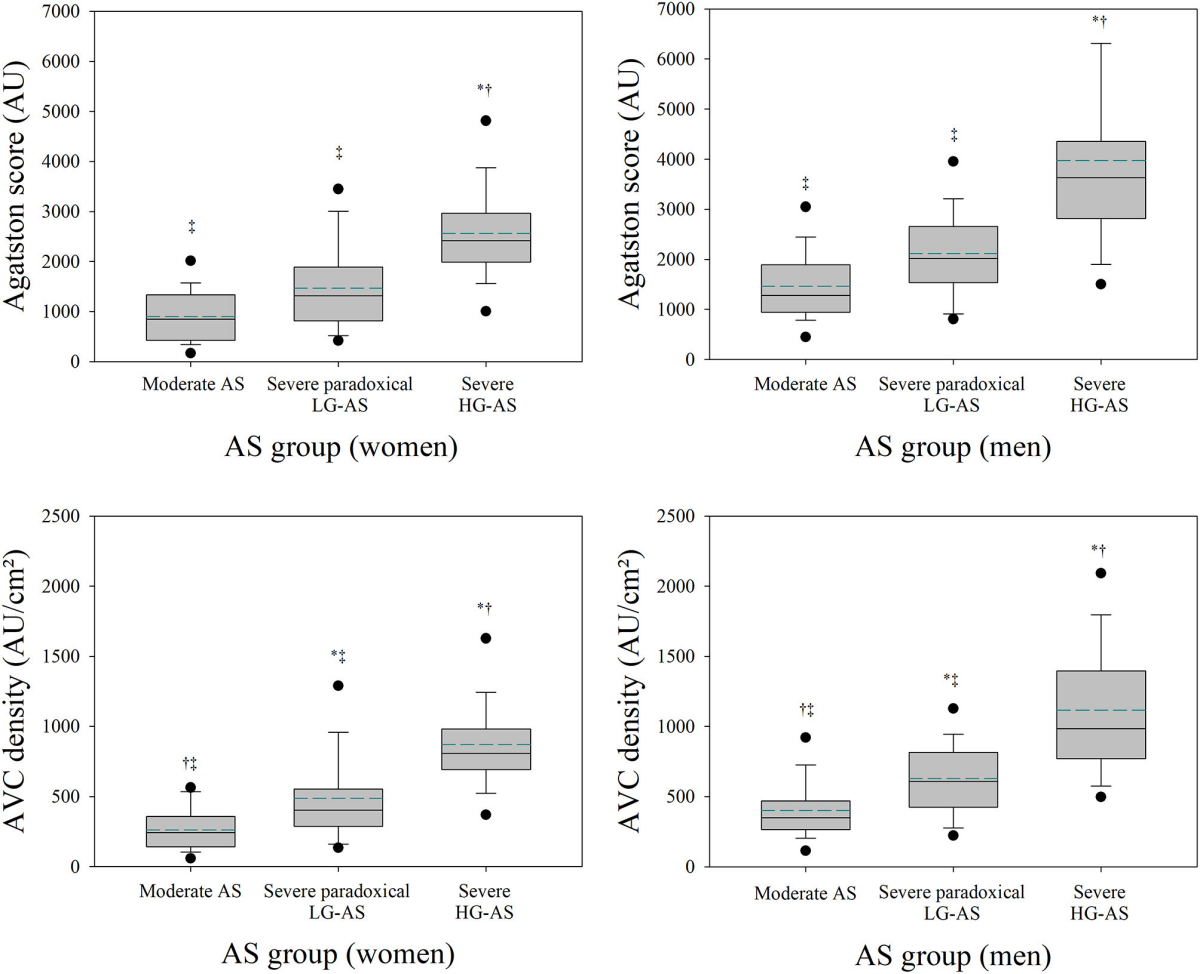
AVC load indices in patients with moderate-to-severe aortic stenosis. Box plots of aortic valve Agatston score (AVC) and AVC density in women (left upper panel) and men (right upper panel) with moderate-to-severe aortic stenosis. The upper and lower edges of the boxes represent the 25th and 75th percentiles. The horizontal line within the boxes represents the median value and the dashed line the mean value. Error bars represent the 95% confidence interval. Pts, patients; AS, aortic stenosis; NF, normal flow; LF, low flow; LG, low gradient; HG, high gradient. *p < 0.05 vs. MAS; ^†^*p* < 0.05 vs. severe paradoxical LG-AS; ^‡^*p* < 0.05 vs. severe HG-AS.

As shown in Table 3 and Figure 2, patients with moderate AS displayed significantly lower Agatston score, AVC density and AVC index than patients with severe HG-AS. Agastson score, AVC density and AVC index of patients with severe paradoxical LG-AS was intermediate between MAS and severe AS. Among patients with severe paradoxical LG-AS, no differences in Agatston score, AVC density or AVC index were found between those with NF and those with LF (Table 3). These observations were made in both men and women, while Agastson score AVC density and AVC index were systematically higher in men than in women.

**Table 3:** AVC load in patients with moderate AS, severe paradoxical LG-AS and severe HG-AS.

	**MAS**	**Severe paradoxical**	**Severe paradoxical**	**Severe HG-AS**	***P-*value**
	**(*n* = 61)**	**NF-LG-AS (*n* = 58)**	**LF-LG-AS (*n* = 47)**	**(*n* = 100)**	
**AVC, AU**	1,185 ± 674^†^^‡^^§^	1,891 ± 951^*^^§^	1,842 ± 888^*^^§^	3,339 ± 1,710^*^^†^^‡^	<0.001
Men	1,462 ± 685^§^	2,190 ± 969^§^	2,027 ± 741^§^	3,975 ± 1,941^*^^†^^‡^	<0.001
Women	900 ± 537^§^	1,435 ± 730^§^	1,516 ± 1,046^§^	2,563 ± 913^*^^†^^‡^	<0.001
**AVC density, AU/cm^2^**	332 ± 192^†^^‡^^§^	543± 278^*^^§^	620 ± 334^*^^§^	1,005 ± 443 ^*^^†^^‡^	<0.001
Men	401 ± 206^‡^^§^	601 ± 304^§^	659 ± 299^*^^§^	1,115 ± 500^*^^†^^‡^	<0.001
Women	206 ± 148^‡^^§^	454 ± 212^§^	551 ± 389^*^^§^	871 ± 319^*^^†^^‡^	<0.001
**AVC index, AU/m^2^**	666 ± 373^†^^‡^^§^	1,021 ± 493^*^^§^	1,004 ± 487^*^^§^	1,815 ± 858^*^^†^^‡^	<0.001
Men	785 ± 389^§^	1,139 ± 518^§^	1,084 ± 413^§^	2,040 ± 975^*^^†^^‡^	<0.001
Women	543 ± 316^§^	845 ± 393^§^	865 ± 585^§^	1,540 ± 593^*^^†^^‡^	<0.001

*Values are mean ± SD. AVC, aortic valve calcium*.

* *p < 0.05 vs. MAS;*

† *p < 0.05 vs. severe paradoxical NF-LG-AS;*

‡ *p < 0.05 vs. severe paradoxical LF-LG-AS;*

§ *p < 0.05 vs. severe HG-AS*.

### MDCT Diagnostic Thresholds in Patients With Concordant AS Grading

As shown in Table 4, using ROC analysis, the best cutoff values to identify severe AS were an Agatston score ≥ 1,577 AU for women and ≥ 2,238 AU for men, an AVC density ≥ 495 AU/cm^2^ for women and ≥ 581 AU/cm^2^ for men and an AVC index ≥ 891 AU/m^2^ for women and ≥ 1,130 AU/m^2^ for men. AVC density was associated with the highest area under the curve (AUC) both for women and men (AUC: 0.98 and 0.96, respectively), followed by the Agatston score (AUC: 0.94 each) and the AVC index (AUC: 0.96 and 0.93, respectivly).

**Table 4:** Diagnostic accuracy of severe calcification in patients with concordant echocardiographic grading.

		**Cutoff**	**Sensitivity (%)**	**Specificity (%)**	**Accuracy (%)**	**AUC**
**AVC (AU)**						
Women	ESC guidelines (17)	1,200	93	66	69	–
	Clavel et al. (18)	1,274	93	73	85	–
	Pawade et al. (22)	1,377	93	83	89	–
	Boulif et al.	1,569	91	90	90	0.94
Men	ESC guidelines (17)	2,000	89	84	87	–
	Clavel et al. (18)	2,065	89	84	87	–
	Pawade et al. (22)	2,062	89	84	87	–
	Boulif et al.	2,238	87	87	87	0.94
**AVC density (AU/cm^2^)**						
Women	ESC guidelines (17)	-	-	-	-	–
	Clavel et al. (18)	292	98	60	83	–
	Pawade et al. (22)	420	93	83	89	–
	Boulif et al.	495	91	90	91	0.98
Men	ESC guidelines (17)	-	-	-	-	–
	Clavel et al. (18)	476	100	77	92	–
	Pawade et al. (22)	527	94	81	89	–
	Boulif et al.	581	87	87	87	0.96
**AVC index (AU/m^2^)**						
Women	ESC guidelines (17)	-	-	-	-	–
	Clavel et al. (18)	637	96	56	80	–
	Pawade et al. (22)	784	91	83	85	–
	Boulif et al.	891	91	90	91	0.96
Men	ESC guidelines (17)	-	-	-	-	–
	Clavel et al. (18)	1,067	89	83	87	–
	Pawade et al. (22)	1,058	89	84	87	–
	Boulif et al.	1,130	87	87	87	0.93

*AUC, area under the curve; AVC, aortic valve calcium*.

### Prediction of Adverse Events by AVC Load Indices

**Figure 3:**
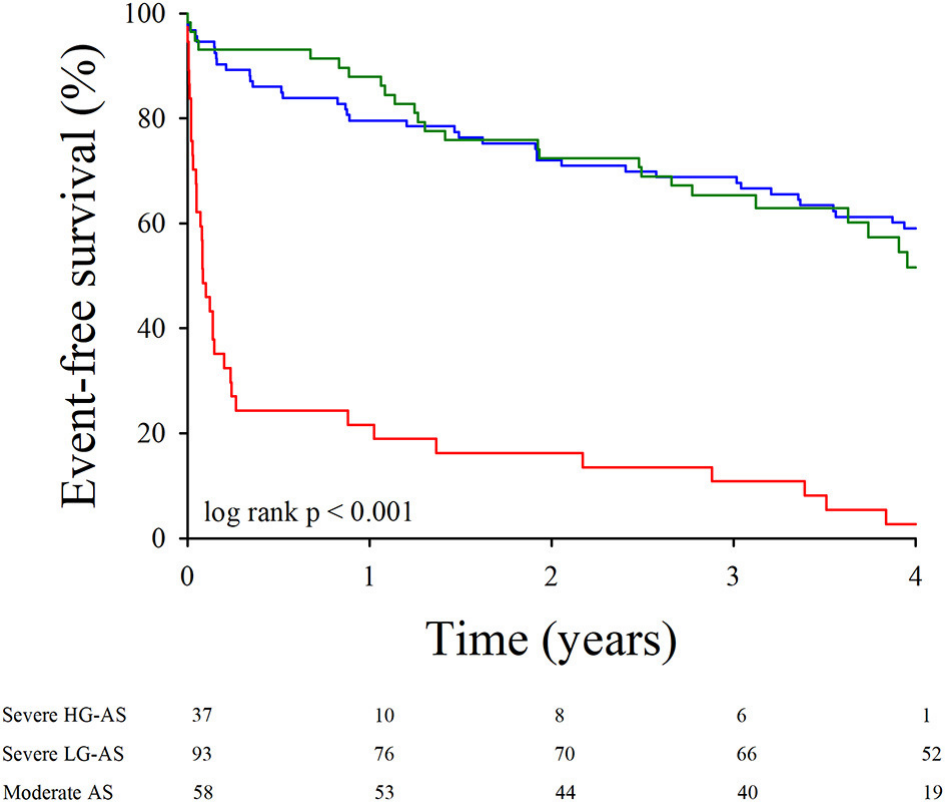
Event-free survival according to baseline AS severity. Kaplan-Meier survival curves for the combined end-point of death and need for aortic valve replacement in patients with severe HG-AS in red, severe paradoxical LG-AS in dark green and moderate AS in dark blue.

Event-free survival was assessed in the 188 patients in whom the decision to proceed to surgery had not yet been made at the time of the MDCT investigation. Over a mean follow-up of 31 months (range 1–48 months), 50 died and 94 underwent aortic valve replacement. The overall event-free survival of this cohort was 72 ± 3%, 63 ± 4%, and 46 ± 4% at, respectively, 1, 2, and 4 years. As shown in Figure 3, event-free survival was better in patients with moderate AS or severe paradoxical LG-AS than in those with severe HG-AS.

**Figure 4:**
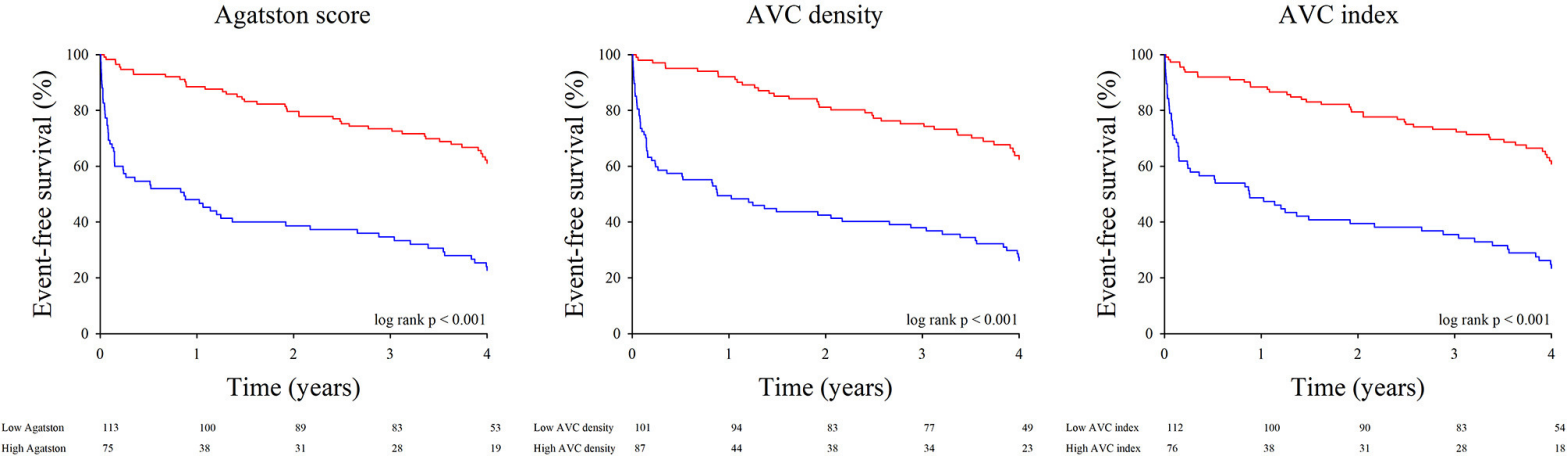
Event-free survival according to AVC load. Kaplan-Meier survival curves for the combined end-point of death and need for aortic valve replacement in patients with low (red line) and high (blue line) AVC load. Left panel: Agatston score; middle panel: AVC density; right panel: AVC index.

**Figure 5:**
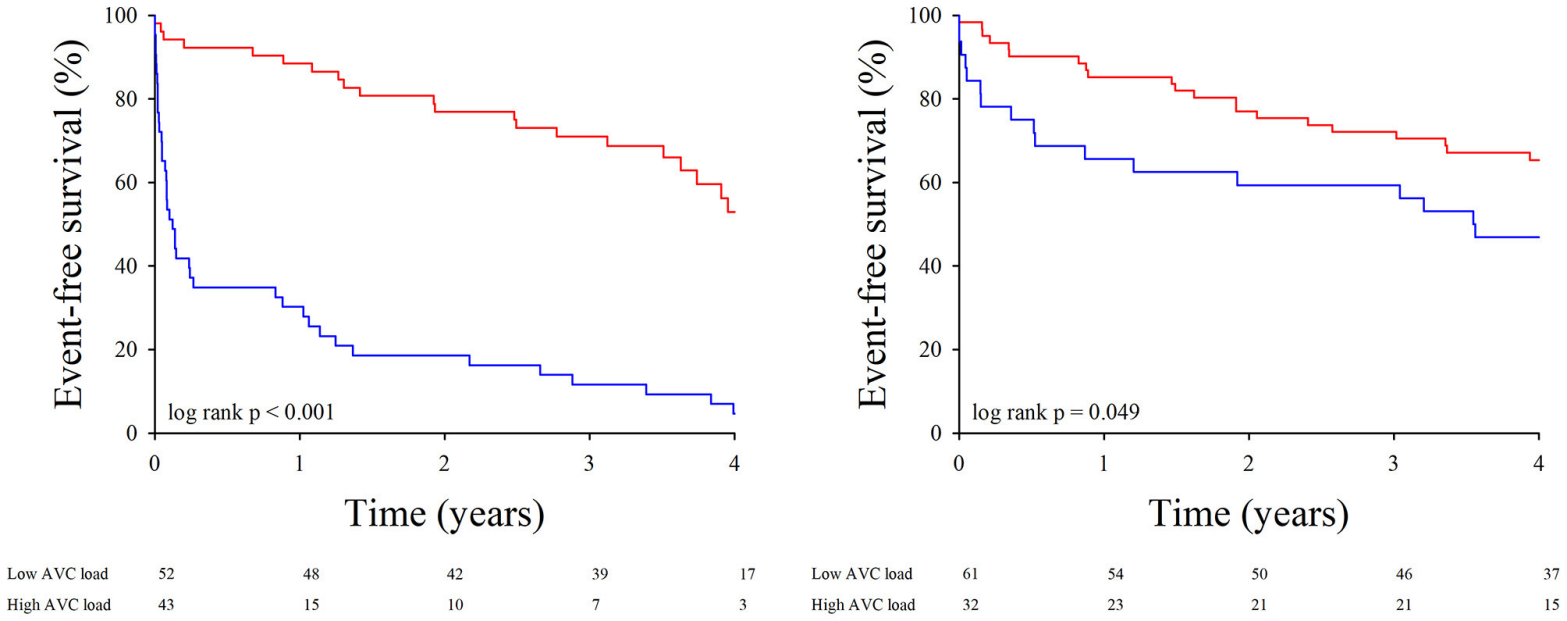
Event-free survival according to AVC load and concordance in AS grading. Kaplan-Meier survival curves for the combined end-point of death and need for aortic valve replacement in patients with in patients with low (red line) and high (blue line) Agatston score. Left panel: patients with concordant AS grading; right panel: patients with discordant AS grading.

The impact of the different AVC load indices on event-free survival was tested in the entire population (Figure 4) as well as in the subgroups of patients with concordant and discordant echocardiographic AS grading (Figure 5). To delineate the factors independently associated with the combined end-point of death and aortic valve replacement, different Cox's proportional hazards regression models were generated. Using AVC load indices as continuous variables (Table 5, model 1), Cox's analysis identified the AVC index as the sole independent predictor of outcome. Using the best AVC load indices cut-off values, as determined by the ROC curve analyses (Table 5, model 2), Cox's analysis identified the AVC Agatston score, age and the effective orifice area as independent predictors of outcome.

**Table 5:** Cox's proportional hazard analysis for the combined end-point of overall mortality and need for aortic valve replacement in the subgroup of 188 patients without a straightaway indication for AVR.

	**Univariate analysis**			**Multivariate analysis**		
**Variable**	**HR**	**95% CI**	***p-*value**	**HR**	**95% CI**	***p-*value**
**Model 1**						
AVC index (/100 AU/m^2^)	1.10	1.08–1.13	<0.001	1.10	1.08–1.13	<0.001
AVC (/100 AU)	1.05	1.04–1.07	<0.001			
AVC density (/100 AU/cm^2^)	1.15	1.10–1.21	<0.001			
Mean gradient (/mmHg)	1.04	1.02–1.05	<0.001			
Peak velocity (/m/s)	2.13	1.65–2.74	<0.001			
Effective orifice area (/cm^2^)	0.16	0.00–0.37	<0.001			
Age (/year)	1.03	1.01–1.05	0.009			
LA dimension (/10 mm)	1.07	1.02–1.14	0.012			
TR <2/4	0.25	0.00–0.87	0.029			
**Model 2**						
High AVC	3.51	2.36–5.24	<0.001	2.82	1.85–4.31	<0.001
High AVC density	3.31	2.20–4.99	<0.001			
High AVC index	3.13	2.10–4.66	<0.001			
Mean gradient (/mmHg)	1.04	1.02–1.05	<0.001			
Peak velocity (/m/s)	2.13	1.65–2.74	<0.001			
Effective orifice area (/cm^2^)	0.16	0.00–0.37	<0.001	0.31	0.00–0.69	0.004
Age (/year)	1.03	1.01–1.05	0.009	1.03	1.01–1.05	0.024
LA dimension (/10 mm)	1.07	1.02–1.14	0.012			
TR < 2/4	0.25	0.00–0.87	0.029			

*LA, left atrium; TR, Tricuspid regurgitation severity*.

### Comparison With Previously Published AVC Load Thresholds

Table 5 compares the sensitivity, specificity and overall accuracy of different AVC load thresholds published in the literature to those found in our study. Overall, the sensitivity of these tresholds was similar (from 91 to 93% in women and from 87 to 89% in men). Differences in specificity were nonetheless observed, the thresholds recommended by the ESC guidelines and those proposed by Clavel et al. being less specific than those proposed by Pawade et al. or those found in the present study.

Using univariate Cox's proportional hazard analyses, we also compared the ability of the different AVC load thresholds to predict outcome. As shown in Tables 6 and 7, all AVC load thresholds were highly predictive of the combined end-point of death and need for aortic valve replamcement. Yet, the model based on the thresholds found in the present study was the most powerful, as shown by its higher χ^2^ and its lower AIC and SBC.

**Table 6:** Prognostic accuracy of severe calcification in patients with concordant echocardiographic grading.

**AVC thresholds**	**HR**	**95%CI**	**χ^2^**	***p-*value**	**AIC**	**SBC**
Clavel et al. (18)	2.27	1.49–3.54	12.82	<0.001	964	967
Guidelines (17)	2.79	1.84–4.25	23.22	<0.0001	953	956
Pawade et al. (22)	2.79	1.86–4.19	24.67	<0.0001	953	956
Boulif et al.	3.51	2.36–5.24	37.99	<0.0001	940	943

*AIC, Aikake Information Criterion; SBC, Schwarz Bayesian Criterion*.

**Table 7:** Prognostic accuracy of severe calcification in patients with concordant echocardiographic grading.

		**Cutoff**	**Sensitivity (%)**	**Specificity (%)**	**Accuracy (%)**	**AUC**
**AVC (AU)**						
Women	ESC guidelines (17)	1,200	65	60	63	-
	Clavel et al. (18)	1,274	61	62	61	-
	Pawade et al. (22)	1,377	56	68	61	-
	Boulif et al.	1,569	54	82	68	0.68
Men	ESC guidelines (17)	2,000	67	69	68	-
	Clavel et al. (18)	2,065	65	73	69	-
	Pawade et al. (22)	2,062	65	73	69	-
	Boulif et al.	2,238	60	80	69	0.70

*AUC, area under the curve; AVC, aortic valve calcium*.

### Proportion of Truly Severe AS in the Different AS Severity Groups

**Figure 6:**
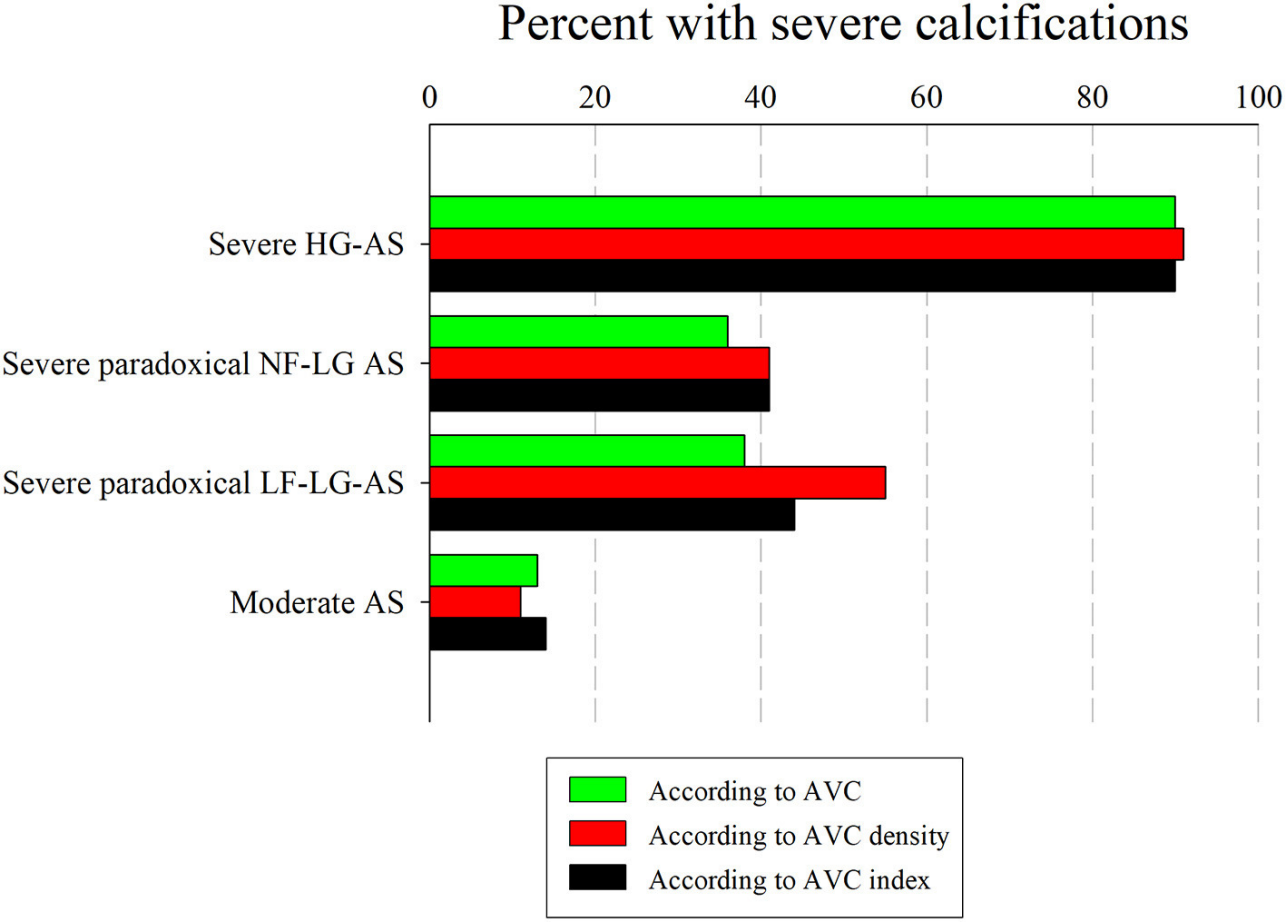
Percent of patients with high AVC load in the different AS severity groups. Percent patients with high Agatston score (in green), high AVC density (in red) and high AVC index (in black) in subgroups with severe HG-AS, severe paradoxical normal flow (NF) or low flow (LF) LG-AS and moderate AS.

**Figure 7:**
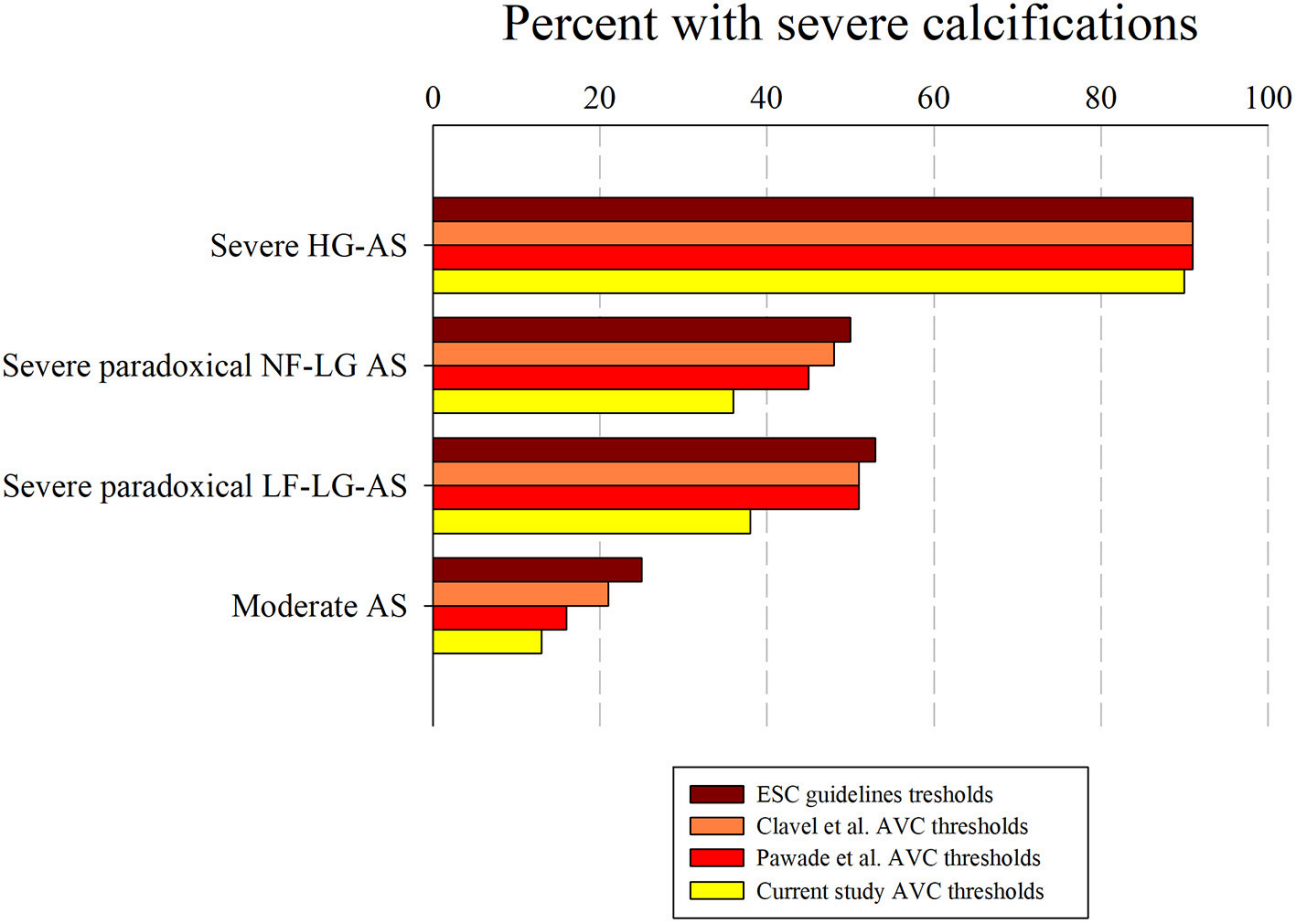
Percent of patients with high AVC load in the different AS severity groups. Percent of patients with high Agatston score according to current thresholds, guidelines thresholds, Clavel's thresholds and Pawade's in subgroups with severe HG-AS, severe paradoxical normal flow (NF) or low flow (LF) LG-AS and moderate AS.

Figure 6 shows the proportion of patients with truly severe AS based on the AVC load thresholds found in our study. As shown, the different AVC load indices correctly identified > 90% of patients with severe HG-AS and > 85% of patients with moderate AS. Depending on the parameter used, 36–55% of patients with severe paradoxical LG-AS also met AVC load criteria for severe AS. Figure 7 shows the same analysis according to the AVC load thresholds proposed in the literature. These thresholds identified a similar proportion of severe calcifications in patients with severe HG-AS. By contrast, all 3 literature thresholds identified a larger proportion of patients with moderate AS displaying severe calcifications. Similar observations were made in patients with severe paradoxical LG-AS.

## Discussion

The aims of the present study were to define, using a standardized MDCT scanning protocol, the optimal AVC load criteria for truly severe AS in patients with concordant echocardiographic AS grading, to establish the potential of these criteria to predict clinical outcomes and to investigate their ability to delineate truly severe AS in patients with discordant echocardiographic AS grading. Our results can be summarized as follows:

- AVC load increases from moderate AS, over severe paradoxical LG-AS to severe HG-AS.
- In patients with concordant echocardiographic AS grading, all different AVC load indices permit to distinguish between moderate AS and severe HG-AS with a similar overall accuracy of 87–91%.
- The observed AVC load thresholds also allow predicting which patient will die or require aortic valve replacement.
- Depending on the criteria used, between 36 and 55% of patients with severe paradoxical LG-AS meet AVC load criteria for severe AS, the lowest proportion being found using our own thresholds, and the highest proportion being obtained using the 2017 ESC guidelines thresholds or those proposed by Clavel et al. and Pawade et al.
- The prognostic impact of AVC load thresholds is less in patients with discordant AS grading than in those with concordant AS grading.

Patients with severe paradoxical LG-AS despite normal LVEF pose diagnostic and management challenges that are distinctly different from the majority of patients with severe HG-AS. Both the European and American guidelines recognize the complexity of reaching a final decision in these patients and consider reasonable to propose AVR in symptomatic patients, provided that clinical, hemodynamic, and anatomic data support valve obstruction as the most likely cause of symptoms (17, 23). According to the most recent ESC guidelines, this can be best achieved by measuring AVC load using MDCT (17).

### AVC Load to Assess the Severity of AS

Earlier studies have shown a definite association between AVC load by EBCT and hemodynamic indices of AS severity (15). More recently, AVC load has also been evaluated with non-ECG gated MDCT (24–26), using a slightly modified Agatston scoring system in order to provide comparable values to the original EBCT quantification. With the introduction of ECG-gating, the quality of cardiac MDCT imaging has improved even further, so that today, MDCT has become the preferred method for assessing AVC load. Its accuracy has been validated in several anatomical studies (16, 26). In the present study, we used this approach to calculate AVC load thresholds that best discriminate between moderate AS and severe HG-AS. Depending on the parameter used (Agatston score, AVC density and AVC index), severe AS was identified with a sensitivity of 87–91%, a specificity of 87–90% and an overall accuracy of 87–91%. Similar results were reported by Clavel et al. (sensitivity of 86–89%, specificity of 80–89%) (18) and Pawade et al. (sensitivity of 80–87%, specificity of 82–84%) (22). Although these last authors found somewhat lower threshold values than in our study, a recent study of Clavel at al. demonstrated that the thresholds of AVC load that best identify adverse outcomes are higher than those proposed in the guidelines and quite similar to those found in the present study (around 1,500 AU in women and 2,250 AU in men) (27).

### Which AVC Load Criteria Should We Use to Assess the Severity of AS?

Since absolute AVC load differs between bicuspid and tricuspid valves, but AVC density does not (16), this latter should probably be preferred to avoid misinterpretations of AVC load in patients in whom the underlying valve morphology is uncertain. The use of AVC density could also avoid underestimation or overestimation of AS severity in patients with small or large annuli, as highlighted by several authors (16, 18, 27). However, current guidelines do not provide any recommendation in this regard. The present study shows that AVC density has the highest accuracy in identifying truly severe AS in patients with concordant AS grading. The thresholds found in our study are also similar to those that were recently found by Clavel et al. as being associated with poor outcomes (430 AU/cm^2^ in women and 560 AU/cm^2^ in men) (27). Further studies are needed to confirm the potential interest in using this parameter instead of the more commonly used Agatston score. Our survival analyses nonetheless suggest that it does allow better prediction of clinical outcomes than the Agatston score.

### AVC Load in Patients With Severe Paradoxical LG-AS

An important finding of this study is that AVC load is significantly lower in patients with severe paradoxical LG-AS than in those with severe HG-AS, irrespective of the flow pattern. It is also higher than in patients with moderate AS. Similar results were reported by Clavel et al. (18) and more recently by Kamperidis et al. (28).

In an earlier analysis of the same cohort, we had already observed that a higher AVC load was needed to define severe AS on the basis of a MPG ≥ 40 mmHg or a Vmax > 4 m/s than on the basis of an EOAi < 0.6 cm^2^/m^2^. We then hypothesized that use of the continuity equation to assess AS severity was responsible for these observations, as the EOAi derived from Doppler echocardiography is usually smaller than the anatomic valve area measured by planimetry, autopsy, or cardiac catheterization. Although the differences between the anatomic and effective valve areas are commonly explained by the continuing convergence of streamlines beyond the anatomical orifice, we have recently shown that in reality, it was largely due to the underestimation of subvalvular flow when inputting a circular LVOT area into the continuity equation (29). Since guidelines for grading AS severity were initially derived from invasive measurements reflecting anatomic valve area, inconsistent grading of AS severity on the basis of mean pressure gradients (or Vmax) and EOAi were to be expected. The present data indicate that use of AVC load might be helpful to better define AS severity, particularly when Doppler echocardiographic data are the most discordant, i.e., in patients with severe paradoxical LG-AS. Indeed, when using the above described AVC load thresholds to define severe AS, 36–55% of patients with severe paradoxical LG-AS meet AVC load criteria for severe AS. These findings are in line with those of Clavel et al. who also found that a substantial proportion (45–53%) of patients with severe paradoxical LG-AS meet AVC load criteria for severe AS (18). This confirms that patients with severe paradoxical LG-AS consist in an heterogenous population, and that use of MDCT to measure AVC load permits to diffrentiate those with truly severe AS from those with moderate or pseudo-severe AS. It should nonetheless be emphasized that the prognostic implications thereof seems to be less in this population than in patients with severe HG-AS. As shown in Figure 4, the event-free survival of patients with severe paradoxical LG-AS and high AVC load is indeed significantly better than that of similar patients with severe HG-AS.

### Study Limitations

This study has limitations that should be acknowledged. First, we had to exclude a significant number of patients from the outcome analyses because they were already scheduled to undergo surgery at the time of their MDCT evaluation. Nevertheless, we were still able to assess clinical outcomes in 188 patients including a large number of patients with concordant or discordant echocardiographic AS grading. Second, we did not perform subgroups analyses in patients with bicuspid vs. tricuspid valves. This is because of the relative inability of echocardiography to accurately identify bicuspid valves when they are heavily calcified (30). This can potentially be problematic when assessing AVC load by use the Agatston score, since bicuspid valves are usually larger than tricuspid valves and therefore tend to display larger Agatston scores than tricuspid valves. As previously shown, this limitation can be easily overcome by use of AVC density instead of absolute AVC score (16). Finally, we did not investigate the potential impact of AVC load on post-operative or -interventional outcomes. Some studies have recently indicated that the presence and amount of calcium in the left ventricular outflow tract was an important determinant of outcomes after transcatheter aortic valve replacement (TAVR) (31). Unfortunately, the small number of patients undergoing TAVR in our study precluded any meaningful statistical analysis. Further studies will be needed to address this issue.

## Conclusions

Assessment of AVC load accurately identifies truly severe AS and provides powerful prognostic information. Our data further indicate that patients with discordant AS grading consist in a heterogenous group, as evidenced by their large range of AVC load. MDCT allows to differentiate between truly severe and pseudo-severe AS in this population as well, although the prognostic implications thereof are not as pronounced as in patients with concordant AS grading.

## Short Summary

Using a standardized MDCT scanning protocol, we identified optimal AVC load criteria for diagnosing truly severe AS. In patients with concordant echocardiographic results, 4-year event-free survival was considerably better with low AVC load by these criteria than with high AVC load. In patients with discordant AS grading, between 36 and 55% of them met AVC load criteria for severe AS. Yet, the prognostic implications thereof was less pronounced than in patients with concordant AS grading.

## Data Availability Statement

The raw data supporting the conclusions of this article can be made available by the authors, without undue reservation.

## Ethics Statement

The studies involving human participants were reviewed and approved by Comité d'éthique hospitalo-facultaire. The patients/participants provided their written informed consent to participate in this study.

## Author Contributions

JB and J-LV have performed the statistical analysis, wrote the initial, and final versions of the manuscript. All authors have contributed to data acquisition, data analysis, and drafting of the manuscript.

## Conflict of Interest

The authors declare that the research was conducted in the absence of any commercial or financial relationships that could be construed as a potential conflict of interest.
